# Nano-Functional Materials for Sensor Applications

**DOI:** 10.3390/molecules29235515

**Published:** 2024-11-22

**Authors:** Aiwu Wang, Li Fu

**Affiliations:** 1 Shenzhen Key Laboratory of Ultraintense Laser and Advanced Material Technology, Center for Advanced Material Diagnostic Technology, College of Engineering Physics, Shenzhen Technology University, Shenzhen 518118, China; 2 College of Materials and Environmental Engineering, Hangzhou Dianzi University, Hangzhou 310018, China

The rapid development of nanotechnology and materials science has led to remarkable advances in sensor applications across various fields [1,2,3,4,5]. Nano-functional materials, with their unique physical and chemical properties at the molecular level, have become increasingly important in designing and fabricating high-performance sensors [6,7,8,9,10,11]. This Special Issue of *Molecules* focuses on the latest developments in nano-functional materials for sensor applications, particularly emphasizing their molecular-level interactions and chemical properties. Over the past decade, the field of nano-functional materials for sensors has experienced significant growth, driven by the increasing demands in healthcare monitoring [12,13,14,15,16], environmental protection [17,18,19,20,21], and security applications [22,23,24,25]. These materials offer unprecedented advantages in terms of sensitivity, selectivity, and response time, making them ideal candidates for next-generation sensing platforms [26,27,28,29,30,31,32,33]. For instance, carbon nanotubes have been integrated into sensors, harnessing their ability to enhance signal transduction and improve detection limits [34,35], while quantum dots have been employed in optical sensors for their high specificity and low detection thresholds [36,37,38].

The molecular-level engineering of these materials has enabled new possibilities in areas such as wearable sensors, biosensors, and environmental monitoring devices [39,40,41,42]. Researchers have leveraged self-assembly and nanoscale fabrication techniques to optimize the material’s interaction with target analytes [43,44,45,46,47,48,49,50]. In healthcare, sensors based on nano-functional materials are being designed to monitor glucose levels without the need for traditional blood draws, providing continuous and non-invasive health metrics [51,52,53]. Similarly, in environmental monitoring, these sensors can detect air pollutants [54,55,56] or waterborne contaminants [57,58,59] with high precision, facilitating early intervention and improved environmental management. These interdisciplinary efforts have led to innovative sensor technologies that address critical challenges in sensor development. Furthermore, advances in materials fabrication have led to the development of sensors that are not only highly sensitive but also cost-effective and scalable, making them suitable for widespread deployment [60,61,62].

This Special Issue contains eleven papers, including two comprehensive reviews and nine research articles, covering various aspects of nano-functional materials in sensor applications. The collected works represent contributions from leading research groups worldwide, offering insights into the current state and future directions of this dynamic field. These papers address critical challenges in sensor development, from molecular recognition mechanisms to practical applications in real-world scenarios. By delving into the molecular-level interactions and chemical properties of nano-functional materials, researchers are paving the way for the next generation of sensors that will revolutionize industries ranging from healthcare to environmental monitoring. The interdisciplinary nature of this research ensures that the advances in sensor technology continue to push the boundaries of what is possible, ultimately leading to better health outcomes, environmental protection, and enhanced security measures.

The two review articles in this Special Issue provide valuable insights into the current developments in sensing applications. The first review by Lu et al. [63] comprehensively discusses recent progress in drug-doping and gene-doping control analysis. This timely review examines various detection methods, including mass spectrometry-based techniques, fluorescence methods, electrochemical approaches, and emerging biosensor technologies. The authors provide a critical analysis of each method’s advantages and limitations, offering valuable guidance for future developments in doping detection. The second review by Zheng et al. [64] presents a detailed analysis of electrochemical sensing methods for detecting lung cancer biomarkers. The authors systematically examine recent advances in nanomaterial-based sensors, conducting polymers, and various recognition elements for detecting specific cancer markers. Their work particularly emphasizes the importance of developing sensitive and selective detection methods for early cancer diagnosis. Both reviews demonstrate excellent scholarship and provide comprehensive overviews of their respective fields, serving as valuable references for researchers working in sensor development and analytical chemistry.

The nine research articles in this Special Issue demonstrate significant advances in the development and application of nano-functional materials for sensing applications. Several papers focus on electrochemical sensing platforms. Xu et al. [65] developed an innovative Fabry–Pérot cavity-based optical fiber sensor using suspended palladium membranes for hydrogen detection, achieving high sensitivity with a detection limit in the ppm range. This work provides valuable insights for developing miniaturized gas sensors. Guo [66] presented a groundbreaking approach involving the production of sea buckthorn juice with pectinase treatment, demonstrating how enzymatic processes can be monitored through electrochemical fingerprinting. Their work offers new perspectives for quality control in food-processing applications. Liu and Shi [67] demonstrated significant advances in using a β-cyclodextrin functionalized platform for monitoring changes in potassium content in perspiration. The research presented an innovative enzymatic method that enabled the real-time monitoring of potassium levels in sweat samples. Their approach showed excellent sensitivity and reproducibility, with practical applications in non-invasive health monitoring. Yu et al. [68] reported a nanoporous-gold-based electrochemical sensor for detecting the anti-tumor drug etoposide in biological samples. Their sensor showed excellent sensitivity and selectivity, with practical applications in therapeutic drug monitoring. Liang et al. [69] developed an in situ derived N-doped ZnO from ZIF-8 for enhanced ethanol sensing in ZnO/MEMS devices. Their innovative approach combined the advantages of metal–organic frameworks and semiconductor materials, resulting in a sensor with improved sensitivity (a response value of 80 towards 25 ppm ethanol) and stability. Deng and Yang [70] developed an innovative silver nanoparticle-embedded hydrogel for the electrochemical sensing of sulfamethoxazole residues in meat. Their approach combines the advantages of hydrogels and metal nanoparticles to create a robust sensing platform for food safety applications. Bianco et al. [71] presented a membrane-based pressure sensor, utilizing advanced materials engineering to achieve precise measurements. Their work demonstrates the potential of integrating nano-functional materials in pressure-sensing applications. Lenar et al. [72] developed an ion-selective electrode for nitrates based on a black PVC membrane, showing how materials engineering at the molecular level can enhance sensor performance. Their approach offers new possibilities for environmental monitoring. Villalonga et al. [73] designed a sandwich-type electrochemical aptasensor with a supramolecular architecture for prostate-specific antigen detection. Their work showcases the integration of molecular recognition elements with nanomaterials for biosensing applications. Each of these contributions demonstrates innovative approaches to sensor development, combining advanced materials science with practical applications. The diverse range of applications—from medical diagnostics to environmental monitoring and food safety—highlights the versatility of nano-functional materials in sensing technologies.

The collection of articles in this Special Issue illustrates the significant advances and versatility of nano-functional materials in sensing applications. From electrochemical biosensors for disease biomarkers and therapeutic drug monitoring, to gas sensors for environmental monitoring and food safety applications, these contributions showcase innovative approaches in materials design, synthesis, and integration. The diverse range of sensing platforms—including MEMS devices, optical fibers, membrane-based sensors, and electrochemical aptasensors—highlights how nano-functional materials can enhance sensitivity, selectivity, and reliability in various sensing applications. The practical demonstrations in real sample analysis further underscore the translational potential of these technologies. We are pleased to announce that the second volume of this Special Issue, “Nano-Functional Materials for Sensor Applications”, is now open for submissions. We welcome high-quality research papers and reviews that address the current challenges and emerging opportunities in the development and application of nano-functional materials for sensing technologies.
